# *MKAN27435* Is Required for the Biosynthesis of Higher Subclasses of Lipooligosaccharides in *Mycobacterium kansasii*

**DOI:** 10.1371/journal.pone.0122804

**Published:** 2015-04-20

**Authors:** Vijayashankar Nataraj, Poh-choo Pang, Stuart M. Haslam, Natacha Veerapen, David E. Minnikin, Anne Dell, Gurdyal S. Besra, Apoorva Bhatt

**Affiliations:** 1 School of Biosciences and Institute of Microbiology and Infection, University of Birmingham, Edgbaston, Birmingham, B15 2TT, United Kingdom; 2 Department of Life Sciences, Imperial College London, London, SW7 2AZ, United Kingdom; Centre National de la Recherche Scientifique - Université de Toulouse, FRANCE

## Abstract

Lipooligosaccharides are glycolipids found in the cell wall of many mycobacterial species including the opportunistic pathogen *Mycobacterium kansasii*. The genome of *M*. *kansasii* ATCC12478 contains a cluster with genes orthologous to *Mycobacterium marinum* LOS biosynthesis genes. To initiate a genetic dissection of this cluster and demonstrate its role in LOS biosynthesis in *M*. *kansasii*, we chose *MKAN27435*, a gene encoding a putative glycosyltransferase. Using Specialized Transduction, a phage-based gene knockout tool previously used to generate null mutants in other mycobacteria, we generated a *MKAN27435* null mutant. The mutant strain was found to be defective in the biosynthesis of higher LOS subspecies, *viz* LOS-IV, LOS-V, LOS-VI and LOS-VII. Additionally, a range of low abundance species were detected in the mutant strain and mass spectroscopic analysis indicated that these were shunt products generated from LOS-III by the addition of up to six molecules of a pentose.

## Introduction

Unique lipids found in the distinct cell wall of mycobacteria are important for integrity and some play a vital role in virulence [1,2]. Lipooligosaccharides (LOS’s) are cell wall associated glycolipids produced by many mycobacterial species including *Mycobacterium canetti*, the ‘smooth’ *Mycobacterium tuberculosis* complex (MTBC) strain [3]. Interestingly, *Mycobacterium tuberculosis*, also a MTBC strain, does not produce LOSs but the genome of *M*. *tuberculosis* H37Rv does contain a ‘reduced’ LOS biosynthetic cluster representative of approximately a third of genes found in *M*. *canetti* [4]. Other LOS producers include the opportunistic pathogen *Mycobacterium kansasii* and poikilotherm pathogen *Mycobacterium marinum* [5,6].

*M*. *kansasii* causes chronic pulmonary and disseminated infections in humans and is the second most common cause of non-tuberculosis mycobacterial (NTM) infections after *Mycobacterium avium* complex (MAC) [7–9]. There are subtle differences in lung lesions caused by *M*. *kansasii* and *M*. *tuberculosis* with regards to the location of lesions and the tendency to form cavitary lesions, and unlike *M*. *tuberculosis*, *M*. *kansasii* infections are not contagious. *M*. *kansasii* is also commonly found in HIV patients; genomic analysis of *M*. *kansasii* strains identified five subtypes associated with HIV patients [10,11]. Mouse infection studies have shown that the rough variant of *M*. *kansasii*, which is deficient in LOS biosynthesis, survives longer causing chronic pulmonary disease compared to smooth, LOS-producing strains [12,13]. Similarly, the LOS-producing MTBC strain *M*. *canetti* is an opportunistic human pathogen, unlike *M*. *tuberculosis* which is virulent in humans. These findings indicate that a loss of LOSs may be one, if not the only, defining factor in the increased virulence of *M*. *tuberculosis* in humans. Furthermore, the role of individual subclasses of LOSs in antigenicity and immunomodulation is poorly understood in context of co-relation between LOS production and severity of disease. Recently, LOS-IV, but not the other LOS-subclasses from *M*. *marinum*, was shown to inhibit TNF-α release in LPS-activated macrophages [14]. Similarly, a LOS-IV-deficient mutant showed increased virulence in infected zebrafish embryos [15]. Furthermore, Alibaud *et al*. [16] observed that *M*. *marinum* strains deficient in LOS production were phagocytized more efficiently than those accumulating only early LOS intermediates, while wild type strains and LOS-IV-lacking mutants were phagocytized with the least efficiency. A fine dissection of role of different LOS-subclasses and their co-relation to the ability to cause disease in models such as *M*.*kansasii* and *M*.*marinum* can eventually help us better understand how *M*. *tuberculosis*, which has lost the ability to make LOS’s through reductive evolution [4], became a more virulent human pathogen than other LOS producing members of the MTB complex like *M*. *canetti*.

**Fig 1:**
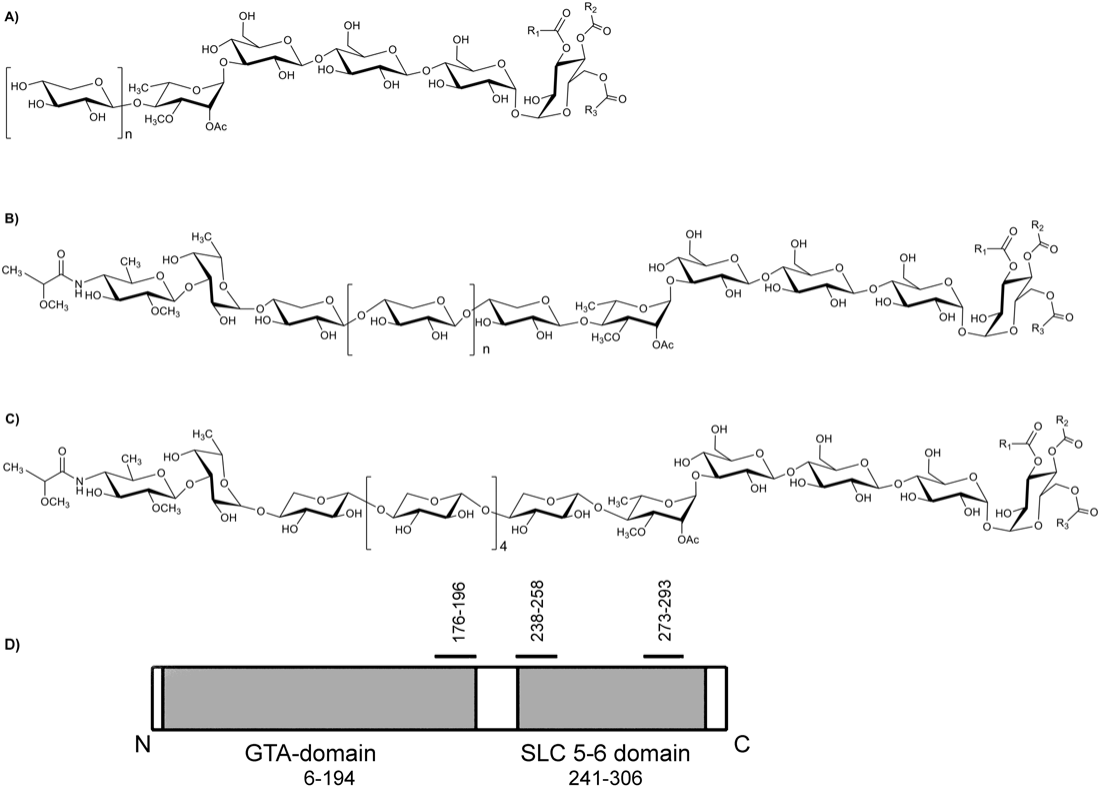
Structures of different LOS subclasses from *M*. *kansasii* (A) LOS-I-III (n = 1–3), (B) LOS-IV-VII (n = 2–4), (C) LOS-VII; R1, R2 and R3 represents the acyl chain attached to tetra glucose core of LOS. (D) Schematic showing predicted domains and topology of *MKAN27435*. The amino and carboxy terminals are indicated as ‘N’ and ‘C’ respectively, and numbers represent predicted borders of domains which are depicted as grey areas. The regions corresponding to the predicted transmembrane domains are indicated with black bars.

*M*. *kansasii* produces seven subclasses of LOS’s, all of which contain a common triacylated, tetra glucose core (Fig 1). In addition all the subclasses of LOSs contain 3-*O*-Me-rhamnose and varying amounts of xylose. Further, LOS subclasses LOS-IV, LOS-V, LOS-VI and LOS-VII contain a terminal fucose and unique sugar *N*-acyl kansosamine [17,18]. While chemical structures of LOS’s from various mycobacteria have been elucidated, far less is known about LOS biosynthesis pathways [17,18]. Most of our understanding of enzymes involved in LOS biosynthesis comes from genetic studies in *M*. *marinum* [4, 19, 20] in which mutant strains accumulating intermediates were used to identify glycosyl transferases involved in LOS biosynthesis. The LOS biosynthesis cluster in *M*. *marinum* includes at least 38 ORFs. By comparing the LOS biosynthesis gene cluster from *M*. *marinum* with the *M*. *kansasii* ATCC12478 genome sequence, we identified a potential LOS biosynthesis cluster in *M*. *kansasii* containing ORFs encoding putative proteins with domains found in glycosyl transferases (GTFs). Targeted deletion of LOS genes will allow us to generate strains that produce intermediate patterns of LOSs, which can be then tested in laboratory models of infection to study the impact of loss of all LOSs, and of individual LOS species, in virulence and immunomodulation. *M*. *kansasii*, with its ability cause opportunistic pulmonary infections in humans, and co-relation of the absence of LOSs to its ability to cause disease, make it a suitable model system to conduct the above mentioned studies. Here, we have generated and characterized a null mutant of the ORF *MKAN27435* which encodes a putative glycosyltransferase, to probe its role in LOS biosynthesis.

## Materials and Methods

### Generation of *MKAN27435* null mutant

The *M*. *kansasii* null mutant Δ*MKAN27435* was generated by Specialized Transduction, a phage mediated gene knockout methodology used previously for other mycobacterial species [21,22]. Approximately 1kb regions upstream and downstream of *MKAN27435* were PCR amplified and cloned on either side of a hygromycin resistance cassette (*hyg*) in the plasmid p0004S [23] to generate the allelic exchange substrate plasmid pΔ*MKAN27435*. pΔ*MKAN27435* was then packaged into the temperature sensitive mycobacteriophage phAE159 [21] to generate the recombinant knockout phage phΔ*MKAN27435*. The wild type (WT) strain *M*. *kansasii* ATCC12478 was transduced with phΔ*MKAN27435* using the protocols described by Larsen *et al*. [23] and the transductants were selected on 7H10 agar plate containing 100 μg/ml hygromycin. Replacement of *MKAN27435* with the *hyg* cassette in the transductants was confirmed by Southern blot (S1 Fig). One such strain was designated Δ*MKAN27435* and was used for all subsequent analysis.

### Complementation of the Δ*MKAN27435* strain

The *MKAN27435* ORF was amplified from *M*. *kansasii* ATCC12478 genomic DNA using the primer pair F27435 (5’-ATGCGAATTCGTGGATTCGGTCAGCGTTG-3’) and R27435 (5’-AT GCAAGCTTTCAGTCCGCCGGATTGTCGAAG-3’). The PCR product was cloned into the *E*. *coli*-mycobacterial replicative shuttle plasmid pMV261 [24] using the primer-incorporated *Eco*RI and *Hin*dIII sites (underlined in primer sequence). The cloned PCR product was verified by sequencing and subsequently electroporated into the Δ*MKAN27435* strain [24]. Transformants were selected on 7H10 plates containing 100 μg/ml hygromycin and 25 μg/ml kanamycin. One such transformant was designated Δ*MKAN27435*-C and was used for subsequent analysis.

### Lipid analysis of *M*. *kansasii* strains

Wild type, mutant and complemented strains *M*. *kansasii* were grown in 10 ml 7H9 broth and lipids were labeled by adding 1μCi/ml [^14^C] acetate (57mCi/mmol) at midlog phase. Apolar and polar lipids were then extracted from the cell pellets obtained from these cultures using methods described by Dobson *et al*. [25]. The extracted lipids were re-constituted in chloroform: methanol (2:1 v/v). The polar lipids were separated by 2-dimensional (2D) TLC using solvent system E [25]. Polar lipids were visualized by autoradiography by exposing the TLC plates to a Kodak BioMax MR film for 24–48 h.

### Purification of LOS’s from *M*. *kansasii* wild type and *ΔMKAN27435*

Cells of *M*.*kansasii* WT and Δ*MKAN27435* strains were harvested from a 6 L culture, and fractions of polar and apolar lipids were extracted using a scaled up version of the protocol of Dobson *et al*. mentioned in the above section [25]. LOS’s were purified from the polar lipid fraction by using DEAE-cellulose an-ion exchange chromatography. Briefly, DEAE-cellulose is equilibrated in chloroform: methanol (2:1 v/v). The polar lipid fraction was applied to the column and eluted with 0, 10mM, 25mM, 100mM and 500mM ammonium acetate in chloroform: methanol (2:1 v/v). The fractions were collected and dried on a rotary evaporator. The dried fractions were reconstituted in chloroform: methanol (2:1 v/v) and separated by 2D TLC using solvent system E [25]. Purified LOS’s on the TLC plates were visualized by α-naphthol staining.

### Mass spectroscopy analysis of LOS subclasses from *M*. *kansasii* WT and mutant strains

The LOS subclasses from *M*. *kansasii* WT and Δ*MKAN27435* strains were permethylated using sodium hydroxide: for each sample, about 5 NaOH pellets were ground to fine powder in a dry mortar with a pestle. About 3 ml of anhydrous DMSO was added to form a slurry. About 1 ml of the resulting slurry was added to the sample before 0.5 ml of methyl iodide was added. The reaction mixture was mixed on a vortex mixer and then agitated on an automatic shaker for 30 min at room temperature. The reaction was quenched with water, while constantly shaking the tube. Permethylated samples were extracted with 1 ml of chloroform and washed several times with 3 ml of water. The chloroform was dried down under a gentle stream of nitrogen gas before sample purification using a Sep-Pak C18 cartridge. The permethylated LOS were then dissolved in methanol before an aliquot was mixed at a 1:1 ratio (v/v) with 10 mg/ml 3,4-diaminobenzophenone in 75% acetonitrile. The glycan-matrix mixture was spotted on a target plate and dried. MALDI-TOF MS data were obtained using a 4800 MALDI-TOF/TOF mass spectrometer (AB Sciex UK Limited) in the positive ion mode. The obtained MS data were viewed and processed using Data Explorer 4.9 (AB Sciex UK Limited).

## Results

### *MKAN27435* encodes a putative glycosyl transferase

The LOS biosynthesis cluster in *M*. *marinum* contains at least 38 genes. In addition to numerous glycosyl transferases, the region also contains a polyketide synthase gene, *pks5* responsible for the biosynthesis of the acyl chains found in LOSs. We used the *M*. *marinum pks5* sequence as a query for a BLAST search to locate a putative LOS biosynthesis cluster in the genome of *M*. *kansasii* ATCC12478 (Table 1). Compared to five LOS-associated glycosyl transferase (GTF) genes found in *M*. *marinum*, the *M*. *kansasii* LOS biosynthetic cluster contains eight genes encoding putative GTFs. Only three orthologues of *M*. *marinum* LOS-associated GTFs are present in *M*. *kansasii*, suggesting that the other GTF genes found in the latter were involved in the addition of the unique sugars found in *M*. *kansasii* LOSs, *viz* fucose and *N*-acyl kansosamine. We then selected *MKAN27435*, a putative GTF-encoding gene that has no orthologues in *M*. *marinum*, for further genetic analysis. BLASTP analysis using the *MKAN27435* sequence revealed matches to LosA (MMAR2313) and WcaA (MMAR2333) from *M*. *marinum*, but with relatively low scores (26% identity and 42% similarity with LosA and 23% identity and 43% similarity with WcaA). A preliminary amino acid sequence analysis of MKAN27435 revealed a domain characteristic of members of the GT-2 family of glycosyltransferases that are similar to eukaryotic dolichol phosphatemannose (DPM) synthases (Fig 1). These GTFs, that typically contain a GT-A fold [26], catalyze the transfer of sugars to dolichol phosphate by using nucleotide sugars as substrates. Topology predictions also indicated the presence of three transmembrane helices, indicating a membrane-associated function. Additionally, the C-terminal region of MKAN27435 contains a domain similar to the solute-binding domain of SLC5 proteins which are Na^+^/sugar co-transporters with affinity for D-galactose, D-glucose, and D-fucose. Given the chemical composition of *M*. *kansasii* LOSs (Fig 1), it seemed likely that MKAN27435 was involved in the addition of either a glucose, or a fucose residue during LOS biogenesis in *M*. *kansasii*. The role could be either direct, involving addition of one of these sugars to a growing LOS intermediate, or indirect, involving the formation of a polyprenyl-linked glucose or fucose moiety that was a substrate for another GTF involved in LOS biosynthesis (S2 Fig).

**Table 1:** Tabulated comparison of genes from LOS biosynthetic clusters of *M*. *kansasii* and *M*. *marinum*.

*M*. *kansasii*	*M*.*marinum*	Putative function
MKAN27390	MMAR2311	Glycosyl transferase, GT-A type
-	MMAR2313	Glycosyl transferase, GT-A type
-	MMAR2333	Glycosyl transferase, GT-A type
MKAN27425	-	Glycosyl transferase, GT-A type
MKAN27430c	-	NAD dependent epimerase/dehydratase family
MKAN27435	-	Glycosyltransferase, GT-A type
MKAN27440	-	Glucose-1-phosphate cytidiltransferase
MKAN27445	-	NAD dependent epimerase/dehydratase family
MKAN27450	-	Rhamnose epimerase
MKAN27475	-	Methyltransferase
MKAN27485	MMAR2340	polyketide synthase
MKAN27490	MMAR2341	Fatty acyl co-A synthatase
MKAN27530	MMAR2342	MmpL family transport protein
MKAN27540	MMAR2344	polyketide synthase
MKAN27575	MMAR2349	Rhamnosyltransferase
MKAN27580	MMAR2351	Glycosyl transferase, GT-A type
MKAN27600	MMAR2353	Glycosyl transferase, GT-B type
MKAN27610	MMAR2355	Acyl transferase
MKAN27675	MMAR2366	Fatty acyl co-A reductase
MKAN27680	MMAR2367	keto acyl reductase
MKAN27695	-	Glycosyl transferase, GT-A type

Where genes are unique to each species, the absence of an ortholog in the other species is indicated by a hyphen (-).

### Loss of *MKAN27435* alters LOS biosynthesis

**Fig 2:**
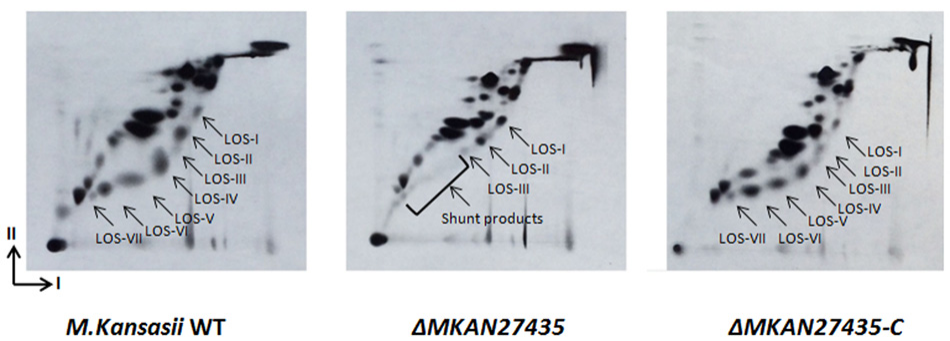
Autoradiograph of 2-D TLC analysis of polar lipids extracted from *M*. *kansasii* WT, Δ*MKAN27435*, Δ*MKAN27435*-C strains. Arrows indicate the different LOS species. I and II indicate system E dimension 1 and 2 respectively. Dimension I: Chloroform: Methanol: H_2_O (60:30:6); Dimension II: Chloroform: Acetic acid: Methanol: H_2_O (40:25:3:6).

Given the presence of *MKAN27435* in a putative LOS biosynthesis cluster, and the outcomes of the bioinformatics analysis described above, we sought to probe the role of *MKAN27435* as a GTF involved in the biosynthesis of LOS’s in *M*. *kansasii*. First, a null mutant of *MKAN27435* was generated in the parental *M*. *kansasii* ATCC12478 strain (referred to as *M*. *kansasii* WT henceforth) using Specialized Transduction. Next, cultures of the strains were grown in the presence of [^14^C]-acetate to label lipids, and LOS’s were isolated as part of the polar lipid fraction from the labeled cultures. Separation of the polar lipids by 2D TLC [25] revealed an altered pattern of LOS species in the mutant strain; while *M*. *kansasii* WT produced all the subclasses of LOS’s (LOS-I, LOS-II, LOS-III, LOS-IV, LOS-V, LOS-VI and LOS-VII) the mutant produced LOS-I, LOS-II and LOS-III, but LOS-IV, LOS-V, LOS-VI and LOS-VII were missing in the strain (Fig 2). Furthermore, a new set of less abundant polar lipid species were detected in the mutant strain (Fig 2). Synthesis of all LOS species was restored in the mutant strain on introduction of a plasmid-encoded copy of *MKAN27435* (Fig 2), indicating that the observed alteration in LOS patterns in the mutant was solely due to the loss of *MKAN27435* function. In addition to the above phenotypes, we did observe a faint spot migrating closest to the origin in the 2D-TLC plate for the *M*. *kansasii* WT strain, which was absent in the mutant strain. However, as the spot did not reappear in the complemented strain, we presumed that this was an artifact and was unrelated to *MKAN27435* function.

### Mass spectroscopic analysis of LOS subclasses

**Fig 3:**
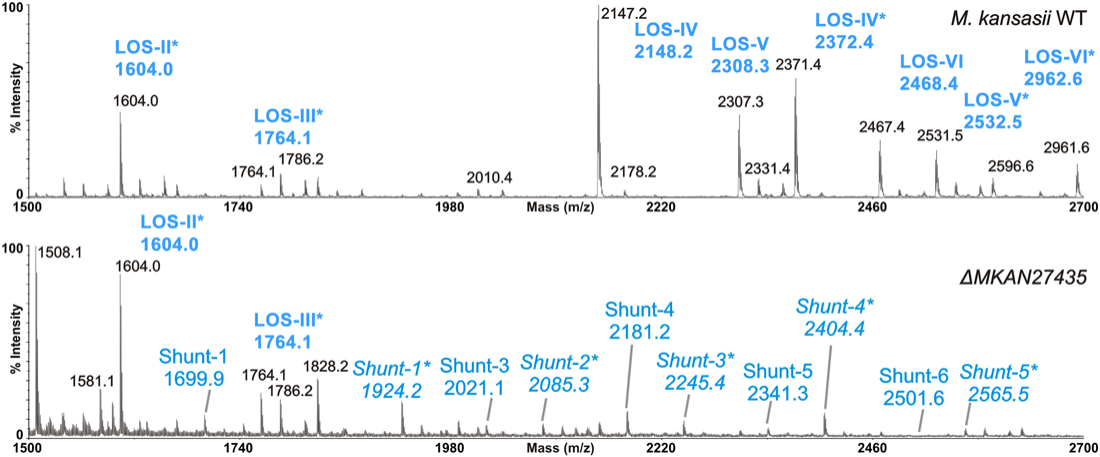
MALDI-TOF mass spectroscopy analysis of purified LOS’s from *M*.*kansasii* WT and Δ*MKAN27435* strains in positive ion mode. All molecular ions are [M+Na]^+^ and species indicated with an asterisk (*) correspond to *m/z* values with an intact acyl group.

To further characterize the set of lower abundance polar lipid species detected in the mutant strain, fractionated polar lipids from the wild type and mutant strains, were subjected to MALDI-TOF mass spectroscopy (Tables 2 and 3; Fig 3). The sample preparation process included a permethylation step, which variably removes acyl groups and replaces them with a methyl group, and also methylates free hydroxyl groups. We were able to thus detect acylated and non-acylated variants. Acyl group retention is indicated by increments of 221Da, for example the species *m/z* 2148.2 corresponds to LOS-IV in the WT strain and *m/z* 2372.4 represents LOS-IV with acyl intact (represented as LOS-IV*). Consistent with the TLC data, higher LOSs were missing in the mutant strain. We also detected a range of low abundance species present only in the mutant strain with sizes that corresponded to incremental additions of *m/z* 160 to LOS-III (Table 3, Fig 3). Each increment corresponded to a per-*O*-methylated pentose and the size ranges observed indicated that the species were likely LOS-III elaborated further with up to six pentose residues.

**Table 2:** MALDI-TOF mass spectroscopy analyses tabulated to list LOS species with corresponding *m/z* values in *M*. *kansasii* WT and Δ*MKAN27435* strains.

*m/z*	LOS species	*M*.*kansasii* WT	Δ*MKAN27435*
1443.91	LOS-I*	+	+
1604	LOS-II*	+	+
1539.9	LOS-III	+	+
1764.1	LOS-III*	+	+
2148.2	LOS-IV	+	-
2372.4	LOS-IV*	+	-
2308.3	LOS-V	+	-
2532.5	LOS-V*	+	-
2468.4	LOS-VI	+	-
2692.6	LOS-VI*	+	-

All molecular ions are [M+Na]^+^and an asterisk (*) indicates species with an intact acyl group. Presence (+) or absence (-) of each species is indicated for each strain.

**Table 3:** MALDI-TOF mass spectroscopy analysis tabulated to list shunt products and corresponding *m/z* values in *M*. *kansasii* Δ*MKAN27435*.

*m/z*	Product	Putative structure
1699.9	Shunt product-1	LOS-III + 1 X pentose
1860.1	Shunt product-2	LOS-III + 2 X pentose
2021.1	Shunt product-3	LOS-III + 3 X pentose
2181.2	Shunt product-4	LOS-III + 4 X pentose
2341.3	Shunt product-5	LOS-III + 5 X pentose
2501.6	Shunt product-6	LOS-III + 6 X pentose
1924.2	Shunt product-1*	LOS-III + 1X pentose
2085.3	Shunt product-2*	LOS-III + 2X pentose
2245.3	Shunt product-3*	LOS-III + 3X pentose
2404.4	Shunt product-4*	LOS-III + 4X pentose
2565.5	Shunt product-5*	LOS-III + 5X pentose

All molecular ions are [M+Na]^+^ and products with an asterisk (*) correspond to *m/z* values with an intact acyl group.

## Discussion

Using Specialized Transduction, we were able to generate the first targeted gene knockout of *M*. *kansasii*, demonstrating the utility of the phage-based method as a genetic tool for this mycobacterial species. The *MKAN27435* null mutant was defective in making four of the seven subclasses of LOS’s indicating that the mutant strain’s inability to extend LOS-III to subsequent LOS subclasses *via* the addition of a fucose residue. Given the presence of domains from DPM-like GTFs in *MKAN27435*, it was likely that MKAN27435 catalyzes the transfer of nucleotide-bound fucose to a polyprenol carrier. Polyprenol-bound fucose could then be ‘flipped’ across the membrane and subsequently utilized as substrate by an extracellular fucosyltransferase to add fucose to LOS-III (S2 Fig). There are a total of two DPM-like GTFs in the *M*. *kansasii* LOS cluster indicating that in part, sugar addition to the LOS core likely occurs *via* polyprenol-linked sugar donors and may thus occur extracellularly. However, there remains an alternative possibility that DPM-like GTFs encoded by LOS clusters directly utilize membrane attached LOS intermediates as substrate.

In addition to the loss of LOS-IV, LOS-V, LOS-VI and LOS-VII, we also observed a number of low abundance polar lipid species in the mutant strain; *m/z* values suggested that these were likely shunt products derived from LOS-III containing up to six additional pentose residues. Though the low abundance of these polar lipid species limited the detailed analysis we could conduct on these species, it could be likely that the pentose residues were additional xyloses transferred to LOS-III in the mutant strain. Surprisingly, we did not observe any difference in the colony morphology of the mutant suggesting that a complete loss of all LOS subclasses was essential for the transition to a rough colony phenotype. The generation and characterization of this mutant in *M*. *kansasii* opens up opportunities for the generation of further targeted mutants in *M*. *kansasii* not limited to the study of LOS biosynthesis.

## Supporting Information

S1 Fig Southern blot confirmation of deletion of *MKAN27435* in *M*. *kansasii* ATCC12478. (A) Schematic representation of the *MKAN27435* region in the *M*. *kansasii* ATCC12478 (WT) genome and its corresponding region in the Δ*MKAN27435* mutant; *HYG*, hygromycin resistance gene from *Streptomyces hygroscopicus*, *sacB*, sucrose counterselectable gene from *Bacillus subtilis*. (B) Individual lanes from a Southern blot of *Kpn*I digested genomic DNA from *M*. *kansasii* WT and Δ*MKAN27435* mutant strains. Probes were the left and right flanking sequences originally PCR amplified to generate the allelic exchange substrate and were labelled using Roche DIG-High Prime DNA labelling and detection kit. Expected restriction bands that bind to the probe are indicated for each strain. Bands were visualised by exposing an X-ray film to chemiluminescence generated as apart of the Southern blotting protocol.
(DOCX)

S2 Fig Schematic showing proposed pathway for LOS biosynthesis in *M*. *kansasii* with regards to the suggested role of MKAN27435. Proposed pathway of LOS-IV biosynthesis in *M*. *kansasii* showing the involvement of MKAN27435 in transferring a nucleotide sugar (fucose) to a polyprenol unit. The polyprenol bound fucose may serve as a sugar donor for the synthesis of LOS-IV by a yet unknown glycosyl transferase. Glu, glucose; Me-Rha, 3-*O*-Me-Rhamnose; Xyl, Xylose; Fuc, Fucose and N-acyl Kan, N-acyl kansosamine.
(DOCX)
