# Supplementary material for: MKAN27435 Is Required for the Biosynthesis of Higher Subclasses of Lipooligosaccharides in Mycobacterium kansasii
Source: PLoS One. 2015 Apr 20;10(4):e0122804. doi: 10.1371/journal.pone.0122804 (PMC4403928; doi:10.1371/journal.pone.0122804)
Supplement: S1 Fig — (A) Schematic representation of the MKAN27435 region in the M. kansasii ATCC12478 (WT) genome and its corresponding region in the ΔMKAN27435 mutant; HYG, hygromycin resistance gene from Streptomyces hygroscopicus, sacB, sucrose counterselectable gene from Bacillus subtilis. (B) Individual lanes from a Southern blot of KpnI digested genomic DNA from M. kansasii WT and ΔMKAN27435 mutant strains. Probes were the left and right flanking sequences originally PCR amplified to generate the allelic exchange substrate and were labelled using Roche DIG-High Prime DNA labelling and detection kit. Expected restriction bands that bind to the probe are indicated for each strain. Bands were visualised by exposing an X-ray film to chemiluminescence generated as apart of the Southern blotting protocol. (DOCX) [file pone.0122804.s001.docx]

**Supporting information S1: Southern blot confirmation of deletion of *MKAN27435* in *M. kansasii* ATCC12478**


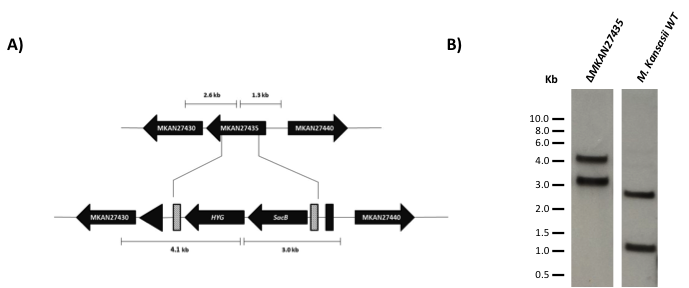


(A) Schematic representation of the *MKAN27435* region in the *M. kansasii* ATCC12478 (WT) genome and its corresponding region in the Δ*MKAN27435* mutant; *HYG*, hygromycin resistance gene from *Streptomyces hygroscopicus*, *sacB*, sucrose counterselectable gene from *Bacillus subtilis*. (B) Individual lanes from a Southern blot of *Kpn*I digested genomic DNA from *M. kansasii* WT and Δ*MKAN27435* mutant strains. Probes were the left and right flanking sequences originally PCR amplified to generate the allelic exchange substrate and were labelled using Roche DIG-High Prime DNA labelling and detection kit. Expected restriction bands that bind to the probe are indicated for each strain. Bands were visualised by exposing an X-ray film to chemiluminescence generated as apart of the Southern blotting protocol.
