# Supplementary material for: MKAN27435 Is Required for the Biosynthesis of Higher Subclasses of Lipooligosaccharides in Mycobacterium kansasii
Source: PLoS One. 2015 Apr 20;10(4):e0122804. doi: 10.1371/journal.pone.0122804 (PMC4403928; doi:10.1371/journal.pone.0122804)
Supplement: S2 Fig — Proposed pathway of LOS-IV biosynthesis in M. kansasii showing the involvement of MKAN27435 in transferring a nucleotide sugar (fucose) to a polyprenol unit. The polyprenol bound fucose may serve as a sugar donor for the synthesis of LOS-IV by a yet unknown glycosyl transferase. Glu, glucose; Me-Rha, 3-O-Me-Rhamnose; Xyl, Xylose; Fuc, Fucose and N-acyl Kan, N-acyl kansosamine. (DOCX) [file pone.0122804.s002.docx]

**Supporting information S2: Schematic showing proposed pathway for LOS biosynthesis in *M. kansasii* with regards to the suggested role of MKAN27435**


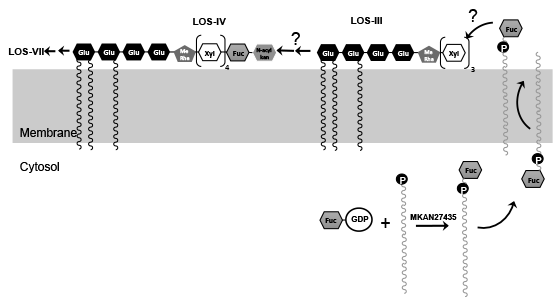


Proposed pathway of LOS-IV biosynthesis in *M. kansasii* showing the involvement of MKAN27435 in transferring a nucleotide sugar (fucose) to a polyprenol unit. The polyprenol bound fucose may serve as a sugar donor for the synthesis of LOS-IV by a yet unknown glycosyl transferase. Glu, glucose; Me-Rha, 3-*O-*Me-Rhamnose; Xyl, Xylose; Fuc, Fucose and N-acyl Kan, N-acyl kansosamine
